# Community Water Fluoridation and Rate of Pediatric Fractures

**DOI:** 10.5435/JAAOSGlobal-D-22-00221

**Published:** 2023-10-05

**Authors:** Sarah E. Lindsay, Spencer Smith, Scott Yang, Jung Yoo

**Affiliations:** From the Oregon Health & Science University, Portland, OR.

## Abstract

**Background:**

The effect of community water fluoridation on bone fragility and fracture has been inconclusive in the literature. The null hypothesis of this study was that no association was observed between water fluoride level and risk of fracture in children.

**Methods:**

Community fluoridation data were obtained from the Centers for Disease Control and Prevention while data on fracture rates were obtained from the PearlDiver database. The rate of fracture type for each state was then compared with state-level fluoridation data using Pearson correlation coefficients and Wilcoxon rank-sum tests.

**Results:**

Positive correlations were found between the percentage of state water fluoridation and fracture rates for both bone forearm fracture (BBFFx) and femur fracture. Fluoride levels had positive correlations with fracture rates for all fracture types. Increased fracture rates were found between states in the highest quartiles of percentage of state water fluoridation and fluoride water levels for supracondylar humerus fracture and BBFFx.

**Conclusions:**

A higher level of water fluoridation was associated with higher rates of supracondylar humerus fracture and BBFFx in children aged 4 to 10 years. These findings do not imply causality, but they suggest that additional investigation into the effect of fluoride on pediatric bone health may be indicated.

Fluoride is an essential microelement known to be crucial in the development of mineralized tissues, including bones and teeth. Fluoridation of community drinking water has been a successful public health measure in preventing the development of dental caries and is one of the most notable public health accomplishments of the 20th century.^1,2^ In the United States, the Centers for Disease Control and Prevention (CDC) monitor community water fluoridation at county and state levels and report these data biannually.

Determining appropriate community water dosing of fluoride has been challenging and controversial. Small amounts of fluoride are essential for mineralized tissues and induce bone formation through the stimulation of osteoblasts and inhibition of osteoclasts.^3^ Too much fluoride is toxic and can cause fluorosis, a condition in which excessive fluoride accumulates in mineralized tissues. Fluorosis is characterized by dental mottling and osteoporosis/osteosclerosis, and, in severe cases, is associated with damage to endocrine, cardiovascular, renal, hepatic, and neurologic organs.^4,5,6,7^ Fluorosis has been identified as a major public health problem, particularly among populations living in areas with high endemic levels of fluoride in water supplies.^8–10^ A 2018 study of residents living in the communities of Northwest Iran found that skeletal fluorosis of people who live in areas with high water fluoride concentrations is 18.1% higher than that of individuals who live in areas with low water fluoride concentrations.^11^ The difference between tolerated dose and toxic dose of fluoride is small and has not been well-established in the literature.^3^ Fluoride is primarily stored in mineralized tissues, and in children, bony retention of fluoride is higher than in adults.^12^ There is mixed evidence regarding exposure to fluoride and adult fractures, with some literature suggesting that fluoride exposure is associated with fragility fractures thought to be due to the deleterious effects of fluoride on bone turnover.^13^ The effects of fluoride on pediatric fracture risk have not yet been assessed.

The purpose of this study was to explore the association between state water fluoride practices and rates of pediatric supracondylar humerus fracture (SCHF) in both bone forearm fracture (BBFFx) and femur fracture (FFx). This study sought to address the gaps that currently exist in the literature regarding the possible association between fluoride and pediatric bone health.

## Methods

### Data

This was an observational, cross-sectional study. A national insurance database (PearlDiver), including both private insurance and Medicaid patients, was queried using Current Procedural Terminology (CPT) codes to identify the number of children aged between 4 and 10 years who were treated for each of the fractures of interest between 2010 and 2020 in each US state. For SCHF, the following CPT codes were used: 24530, 24535, 24538, 24545, and 24546. For BBFFx, the following CPT codes were used: 25560, 25565, 25574, and 25575. For FFx, the following CPT codes were used: 27500, 27502, 27506, and 27507.

2020 US Census data were then obtained to identify the number of children in each age group in each state.^14^ Based on these data, an estimated annual rate was calculated for each fracture type in each state.

Centers for Disease Control and Prevention (CDC) data were used to determine the percentage of each state's population receiving fluoridated water.^15^ Percentages were reported by the CDC in 2-year intervals, and data from 2010, 2012, 2014, 2016, 2018, and 2020 were averaged to yield a percentage of each state's population receiving fluoridated community water for this study's 10-year period. Data from all 50 states were available.

We determined the weighted average of states' fluoride level by multiplying the fluoride level in each county by the population of that county divided by the total state population.^15^ Dosage data were available for 31 states. Available data since 2015 were collected and averaged to yield a 5-year average fluoride level per state.

### Statistics

Pearson correlation coefficients were calculated for the relationships between state-level fracture rates and state percentage of fluoridated water. Pearson correlation coefficients were also calculated for the relationships between state-level fracture rates and average state-level water fluoride levels for each fracture type in the 31 states with available fluoride level data. Significance levels were determined.

Wilcoxon rank-sum tests were used to compare state percentage of fluoridation by quartiles with state-level fracture rates. We compared the differences in fracture rates between the highest and lowest quartile percentage state fluoridation groups for each fracture type. Quartiles were also calculated based on the average state fluoride level, and differences in fracture rates sustained by those in the highest and lowest quartiles were determined.

## Results

In total, 106,423 pediatric patients were identified in the PearlDiver database who met the inclusion criteria for this study for diagnosis, date of injury, and age: 40,197 SCHF patients, 61,041 BBFFx patients, and 5,185 FFx patients. For all fracture types, the average age was between 6 and 7 years (Table 1).

**Table 1:** Demographics for Fracture Cohorts

	Supracondylar Humerus Fracture (SCHF)	Both Bone Forearm Fracture (BBFFx)	Femur Fracture (FFx)
n	40,197	61,041	5,185
Age, mean (SD)	6.30 (1.69)	6.91 (1.87)	6.44 (2.03)
Sex, % female	47.5	40.6	32.2
Region			
Midwest, n (%)	9,882 (24.6)	15,797 (25.9)	1,400 (27.0)
Northeast, n (%)	5,792 (14.4)	9,512 (15.6)	835 (16.1)
South, n (%)	17,808 (44.3)	26,455 (43.3)	2,138 (41.2)
West, n (%)	6,335 (15.8)	8,762 (14.4)	742 (14.3)

The average percentage of state population receiving fluoridated water ranged from 10.6% (Hawaii) to 100% (Washington DC) with a median of 76.94% (first quartile: 57.62%, fourth quartile: 91.52%). In most states (84%), more than 50% of the population received fluoridated water. In the 31 states with county-level data on fluoride levels, the average state fluoride level ranged from 0.39 to 0.7 mg/L (Table 2).

**Table 2:** Average Percent of State Receiving Fluoridated Water (%) and Average Fluoride Level (mg/L) For Each State and Fracture Type

State	Average Percentage of State Receiving Fluoridated Water (2010–2020), %	Average Fluoride Level (mg/L)	SCHF Fracture Rate per 100,000 Children Aged 4–10 yrs	BBFFx Fracture Rate per 100,000 Children Aged 4–10 yrs	FFx Fracture Rate per 100,000 Children Aged 4–10
Alabama	78.48	0.56	4.79	12.86	0.30
Alaska	51.05	0.39	4.43	7.06	0.00
Arizona	57.63		21.37	29.81	3.72
Arkansas	76.45	0.60	5.32	11.01	0.48
California	61.15	0.42	4.47	5.28	0.33
Colorado	73.58	0.65	12.27	18.24	1.25
Connecticut	90.03	0.63	7.70	9.23	0.57
District of Columbia	96.00		7.64	8.42	0.00
Delaware	88.82	0.55	5.93	16.92	1.36
Florida	77.82	0.55	17.34	26.34	1.86
Georgia	95.15	0.66	14.40	25.91	1.92
Hawaii	10.25		14.06	8.51	0.00
Idaho	32.30		3.83	6.54	0.00
Illinois	98.57	0.70	11.38	17.14	1.10
Indiana	93.83	0.67	18.09	29.32	2.50
Iowa	91.23	0.69	17.48	35.14	2.05
Kansas	64.85	0.53	11.43	17.47	1.01
Kentucky	99.88	0.70	23.29	38.66	2.13
Louisiana	41.58	0.43	13.51	23.96	2.56
Maine	79.30	0.56	18.03	27.10	2.37
Maryland	95.70		14.90	33.59	1.61
Massachusetts	64.08	0.44	5.56	9.56	0.62
Michigan	90.33	0.64	23.35	34.23	3.61
Minnesota	98.80	0.69	7.61	14.65	1.56
Mississippi	59.37	0.51	6.92	15.36	0.97
Missouri	76.27	0.55	9.03	19.02	1.36
Montana	31.85		4.56	7.17	0.00
Nebraska	71.67	0.59	12.73	25.57	1.32
Nevada	74.42	0.60	9.23	17.30	1.10
New Hampshire	45.82	0.45	4.86	11.95	0.00
New Jersey	14.93		17.17	28.39	2.26
New Mexico	76.92		16.85	29.09	2.07
New York	72.10		14.61	23.09	1.99
North Carolina	87.63		6.41	13.07	0.84
North Dakota	96.52	0.67	11.52	19.25	0.00
Ohio	91.72		26.54	39.44	4.23
Oklahoma	67.08		14.07	23.49	1.56
Oregon	23.85		19.05	25.60	2.39
Pennsylvania	55.68		11.86	20.67	2.27
Rhode Island	84.02	0.59	4.58	7.68	0.00
South Carolina	91.58	0.70	18.03	34.91	3.80
South Dakota	93.85		20.00	32.57	2.68
Tennessee	89.23	0.63	20.65	27.30	2.62
Texas	75.05	0.60	21.94	21.51	2.32
Utah	48.73		5.61	12.14	0.65
Vermont	56.28		0.00	3.29	0.00
Virginia	95.93	0.70	10.80	24.48	1.63
Washington	64.17	0.60	15.52	20.82	1.80
West Virginia	90.98		20.25	41.21	2.96
Wisconsin	88.08		13.54	21.28	1.95
Wyoming	51.22		8.37	13.31	0.00

BBFFx = both bone forearm fracture, FFx = femur fracture, SCHF = supracondylar humerus fracture.

**Figure 1:**
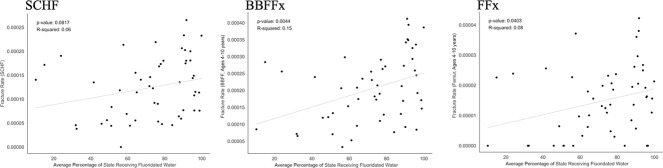
Graphs with correlation analyses demonstrating positive correlations between state percentage fluoridation and fracture rates for BBFFx (*r* = 0.39, *P* = 0.0044) and FFx (*r* = 0.28, *P* = 0.040). Although a positive association was demonstrated for SCHF (*r* = 0.24), this association was not significant (*P* = 0.082). BBFFx = both bone forearm fracture, FFx = femur fracture, SCHF = supracondylar humerus fracture.

In our correlation analysis, we found positive correlations between state percentage fluoridation and fracture rate for BBFFx (*r* = 0.39, *P* = 0.0044) and FFx (*r* = 0.28, *P* = 0.040). Although a positive association was demonstrated for SCHF (*r* = 0.24), this association was not significant (*P* = 0.082) (Figure 1).

**Figure 2:**
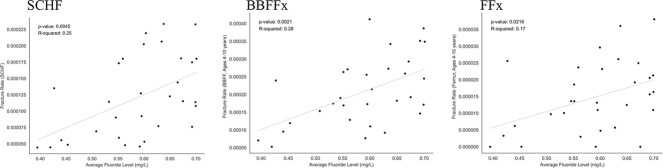
Graphs with correlation analyses demonstrating positive correlations between the calculated concentrations of fluoride per liter of drinking water based on weighted averages of fluoride concentration by county population and fracture rates for SCHF (*r* = 0.5, *P* = 0.0045), BBFFx (*r* = 0.53, *P* = 0.0021), and FFx (*r* = 0.41, *P* = 0.022). BBFFx = both bone forearm fracture, FFx = femur fracture, SCHF = supracondylar humerus fracture.

The calculated concentrations of fluoride in per liter of drinking water based on the weighted averages of fluoride concentration by county population had strong positive correlations with fracture rates for all fracture types: SCHF (*r* = 0.5, *P* = 0.0045), BBFFx (*r* = 0.53, *P* = 0.0021), and FFx (*r* = 0.41, *P* = 0.022) (Figure 2).

**Figure 3:**
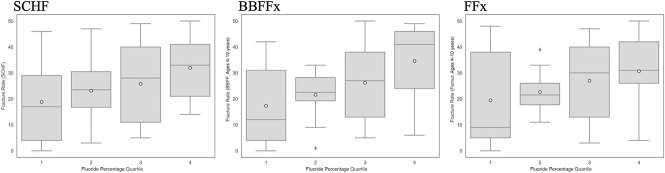
Graphs with Wilcoxon rank-sum analyses demonstrating that fracture rates significantly differed between the highest quartile and the lowest quartile fluoridation percentage states for SCHF (*P* = 0.0428) and BBFFx (0.0052). The rates for femur fractures did not significantly differ (*P* = 0.16). BBFFx = both bone forearm fracture, SCHF = supracondylar humerus fracture.

Fracture rates significantly differed between the highest quartile and the lowest quartile fluoridation percentage states for SCHF (*P* = 0.0428) and BBFFx (0.0052). Rates for FFx did not significantly differ (*P* = 0.16) (Figure 3).

**Figure 4:**
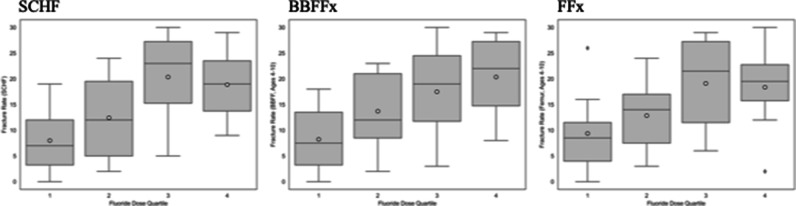
Graphs with Wilcoxon rank-sum analyses demonstrating that fracture rates significantly differed between the highest quartile and lowest quartile average state fluoride levels for SCHF (*P* = 0.012) and BBFFx (0.012). The rates for femur fractures did not significantly differ (*P* = 0.059). BBFFx = both bone forearm fracture, SCHF = supracondylar humerus fracture.

Fracture rates significantly differed between the highest quartile and lowest quartile average state fluoride levels for SCHF (*P* = 0.012) and BBFFx (0.012). No significant differences were found for FFx (*P* = 0.059) (Figure 4).

**Figure 5:**
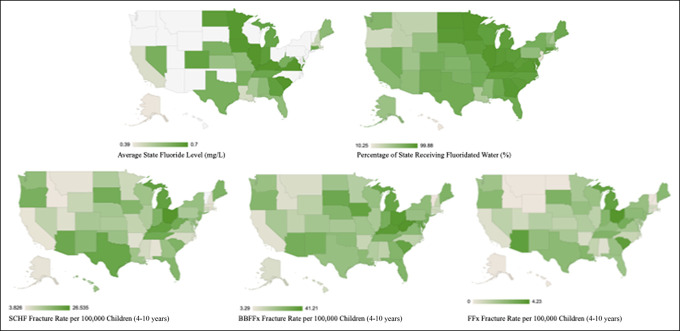
Illustrations of US heat maps depicting relative average state fluoride levels (mg/L), percentage of state population receiving fluoridated water (%), and rates of each fracture type by state.

Heat maps were generated to visually demonstrate relative average state fluoride levels (mg/L), percentage of state population receiving fluoridated water (%), and rates of each fracture type by state (Figure 5).

## Discussion

This study is the first to explore the association between community water fluoridation and rate of pediatric fractures using national databases. In our analysis of the PearlDiver cohort of 106,423 pediatric patients with SCHF, BBFFx, or FFx aged between 4 and 10 years, we found statistically significant associations between fracture rate, state-level percentages of population using fluoridated water, and calculated fluoride doses. To our knowledge, this is the first study investigating the community water fluoridation and its possible association with pediatric fracture risk.

Fluoride is well-established as an essential nutrient necessary for the development of bones and teeth. It plays a crucial role in the modulation of bone turnover through its effects on osteoblasts and osteoclasts. At low doses, fluoride increases bone mass through the inhibition of osteoclasts and the promotion of osteoblasts.^16^ A study on cell lines found that fluoride induces epigenetic changes through DNA hypermethylation in BMP1, METAP2, MMP11, and BACH1, which, in turn, may affect cartilage formation, angiogenesis, and bone density.^17^

The physiologic effects of fluoride seem to be dose-dependent. High doses of fluoride are associated with delayed bone mineralization and decreased bone mechanical properties.^18^ In vitro treatment of rat ulnas with sodium fluoride resulted in decreased bone turnover and diminished mechanical properties (elastic modulus, ultimate stress, and bending rigidity).^19^ In sheep, high fluoride (9.8 mg/L) intake from groundwater combined with low calcium led to poor bone quality and fragility fractures.^20^ In humans, excessive fluoride intake results in skeletal fluorosis. It is hypothesized that, at higher doses, fluoride inhibits normal bone turnover, and although bone volume is increased, trabecular connections are not, resulting in poor-quality, weak bone.^3,21^

Fluoridation of community water has been studied extensively because of the potentially harmful effects of excessive fluoride intake. In 2015, the US Public Health Service updated its 1962 recommendations to recommend an optimal community fluoride water concentration of 0.7 mg/L, with a maximum permitted concentration of 4 mg/L.^22^ The World Health Organization recommends 1.5 mg/L.^23^ These recommendations are often debated and are frequently revisited with the goal of maximizing benefits of fluoride intake for bone and teeth health while minimizing harm.

The effect of fluoride in drinking water on adult hip fractures has been studied extensively, although the results of these studies have been inconclusive. In 2013, a large Swedish cohort study found that long-term exposure to fluoride of 2.7 mg/L was not associated with hip fracture.^24^ A 2015 meta-analysis found that chronic exposure to fluoride in drinking water did not markedly increase the risk of hip fractures in adults.^25^ Other work has found fluoride to increase bone mineral density in adults and low doses of fluoride (</ = 20 mg/d) to be protective against vertebral and hip fractures.^26^ However, a 2021 Swedish study of postmenopausal women found that chronic low-dose fluoride exposure (<1.5 mg/L) was associated with both increased bone mineral density and fragility fractures.^13^

There has been little research investigating the effects of fluoride on bone in the pediatric population.^27^ It has been established that children retain more fluoride in bones than adults do, but the implications of this are unknown.^12^ Previous work has shown a relationship between skeletal fragility and fracture risk in children.^28^ Developing a better understanding of the effect of community water fluoridation on pediatric bone health could better inform public health guidelines in the future, such as adjusting recommended water fluoride levels or providing a basis for the recommendation of calcium supplementation.

The major limitation to this study relates to inferences of the population based on sampling. The PearlDiver database is a randomly selected sampling of Medicaid and most private insurance companies. Although the database likely represents an adequate cross-sectional sampling of all pediatric patients, one weakness of the study is a sampling error resulting in different capture rates of children with different insurance types. Similarly, the data inferred from the US Census Bureau and CDC are prone to sampling error. Despite these limitations, we think that the retrospective use of best available representative large databases can explore population-based questions. Our study design resembles a natural experiment design and is useful when an exposure of interest is not possible to be assigned to research subjects, either practically or ethically. In evaluating the effect of fluoridation, a control group cannot be created easily because most communities are already exposed to fluoridated water sources. Furthermore, a prospective randomized study cannot be done when a condition is rare (177.3 per 100,000 population), and a notable portion of the population is already receiving fluoridated water. It is important to recognize that SCHF data were available only at the state level and there was substantial variation in fluoride rates by county, making it somewhat difficult to draw conclusions based on our data.

The constraints in our database prevent us from specifically studying or excluding children with malnutrition, malabsorption, bone disease, or other chronic disease. We are also unable to identify those who take calcium or vitamin D regularly. Pediatric bone health is a complex, nuanced topic, and a notable limitation of this study is a lack of granularity regarding other factors that may contribute to pediatric fragility fractures. In addition, we do not have the ability within this database to classify whether fractures sustained were fragility fractures, although this would be an interesting area of future study.

Previous work has established that the effects of fluoride on bone and other tissues are dependent on genetic factors.^29–31^ Although genetic susceptibility to fluoride exists, we think that the diverse population base in the United States minimizes these effects compared with studying a small region of the world where a more homogeneous genetic composition might be expected. In addition, many factors influence bone density and health, including other important nutrients, activity level, and genetics. Because this is a large population-based study based on administrative data, a multivariate analysis of all possible bone health factors is not feasible. However, because it is a large population-based study sampling Medicaid and private insurance population in United States, we expect random distribution of these other factors.

We propose simply an association worth considering as both percentage and dose-dependent concentration of fluoride are associated with fracture rate. We note that measured fluoride levels in community drinking water do not indicate fluoride levels in a patient, and we do fully capture levels of fluoride exposure in this study. We do not imply causality based off of these findings, and we suggest that additional studies be done to investigate this possible relationship between pediatric bone health and fluoride. Although there are numerous studies on fluoridation and effects of fracture on adults, very few studies address children's bone health. It is possible that growing bone will have a different response to fluoride intake compared with adult bone. In this study, we present a novel approach in examining this epidemiologic question at a national level with a common pediatric fracture type.

## Conclusions

In the PearlDiver data cohort, community water fluoridation proportion by both state and fluoridation levels are associated with the increased rate of fracture in children aged 4 to 10 years. This research suggests that more studies are needed to further define issues such as correlation with other fractures and determination of the critical level of fluoridation in growing bone.
